# Deep learning–enhanced clustering and classification of protein molecule tertiary structures using weighted distance matrices

**DOI:** 10.1093/bib/bbaf331

**Published:** 2025-07-08

**Authors:** Junlong Liu, Jiaming Xiao, Xunwen Su, Yonglin Wang

**Affiliations:** School of Technology, Beijing Forestry University, 35 Qinghua East Road, Haidian District, Beijing 100083, China; School of Technology, Beijing Forestry University, 35 Qinghua East Road, Haidian District, Beijing 100083, China; School of Technology, Beijing Forestry University, 35 Qinghua East Road, Haidian District, Beijing 100083, China; National Facility Preservation Bank for Forestry and Grassland Germplasm Resources, Beijing Forestry University, 35 Qinghua East Road, Haidian District, Beijing 100083, China; State Key Laboratory of Efficient Production of Forest Resources, College of Forestry, Beijing Forestry University, 35 Qinghua East Road, Haidian District, Beijing 100083, China

**Keywords:** Alphafold2, clustering algorithm, unsupervised classification

## Abstract

Protein clustering and classification are critical for understanding protein functions and interactions, particularly within structure-based predictions. Traditional sequence-based clustering often overlooks the pivotal role of tertiary structure in determining protein function. Structural clustering remains limited and challenging, with existing methods struggling to achieve high accuracy and manage complex data. This study focuses on the tertiary structures of *Verticillium dahliae* proteins, employing deep learning techniques for effective clustering and classification. Using AlphaFold2, we predicted protein structures and generated Cα atom distance matrices. We introduced a novel Unique Nuclear Sequence Element (UNSE) neural network to enhance feature extraction, constructing weighted distance matrices by integrating Cα distances with Pfam annotations. This method effectively captures complex structural relationships. Additionally, Basic Local Alignment Search Tool (BLAST) sequence alignments validated the sequence similarity within protein families, ensuring the biological relevance of clustering results. We applied clustering algorithms to both raw and weighted matrices, comparing their performance against traditional sequence-based and other structure-based methods, including DeepGO and DeepFRI. Evaluation metrics such as Silhouette Score, ${F}_{max}$, and AUPR demonstrated that our weighted matrix approach significantly outperforms conventional methods in accuracy and robustness. These findings confirm that integrating deep learning with weighted distance matrices effectively captures structural and functional protein characteristics, providing a robust tool for structural biology.

## Introduction

The 3D structure of proteins is pivotal to their biological functions and interactions, significantly influencing their activity [1–3]. Unlike the linear primary sequence, the tertiary structure defines the precise spatial arrangement of a protein, determining its physical, chemical, and functional properties within biological systems [4, 5]. Research by Listov *et al*. has shown that tertiary structure provides a more reliable basis for identifying functional similarities between proteins than primary sequences [6]. Therefore, studying the clustering and classification of tertiary structures holds great potential for enhancing our understanding of protein function and biological significance [7].

Current protein clustering and classification methods are mainly categorized into sequence-based methods, structure-based methods, and hybrid methods. Current methodologies predominantly depend on sequence similarity metrics for initial protein classification. Such methods work under the premise that proteins with like sequences are typically also functionally similar such as convolutional neural network (CNN)–based sequence comparison methods [8–11] or hierarchical clustering methods using BLAST [12]. However, methods that rely solely on sequence information have limitations in dealing with complex protein structure–function relationships. For example, Tsuyuzaki *et al*. [13] proposed a sequence clustering method that combines Principal Component Analysis (PCA) with traditional clustering algorithms, and although it performs well in sequence clustering performance, it cannot deal with protein tertiary structures. In addition, sequence-driven tools (e.g. BLAST and MMseqs2) utilize sequence comparisons to infer homology but are unable to cluster proteins with similar functions but low (<30%) sequence identity, such as T-cell immunoglobulin and mucin domain (TIM) tubulin, which has the same catalytic mechanism but different sequences. With the advancement of 3D-CNNs, researchers have been progressively using them to predict the functional sites of proteins. The accuracy and robustness of the model are enhanced by the superior capability of 3D-CNNs to capture biological characteristics in protein 3D structures [14, 15], compared to 2D convolutional networks and conventional sequence comparison methods. However, Gao pointed out [16] that although 3D-CNNs perform well in function prediction, reducing computational complexity and improving the interpretability of the model remain challenges. There is mounting evidence, as studies have developed [17, 18], that sequence similarity is insufficient on its own to capture the functional variety of proteins properly. Specifically, sequence similarity might not be able to reliably predict the biological roles of proteins that operate similarly but with low homology [19]. On the other hand, the spatial conformation and its relationship to function can be more clearly reflected in the tertiary structure of proteins. Structure-centered methods (e.g. Foldseek, TM-align) use structural comparisons (e.g. TM-score) to determine distal homology, and AlphaFold2 allows large-scale structural clustering of 2.3 million unannotated folds. Researchers can capture key structural changes that are closely related to function by incorporating the spatial characteristics of tertiary structures [20]. However, these approaches face computational bottlenecks (O(n [2]) complexity) and tend to ignore dynamic conformations that are crucial for heterodimer regulation. Hybrid strategies aim to bridge this gap: while InterProScan integrates Pfam structural domains and structural matrices but relies on static databases, DeepFRI employs graph neural networks (GNNs) to map sequence structure embeddings to functional terms, achieving 89% accuracy in enzyme prediction. Despite progress, key limitations remain: sequence-structure decoupling, scalability–resolution tradeoffs, and poor interpretability of deep learning models. Thus, developing algorithms that integrate both sequence and structural information is pivotal for enhancing protein function prediction accuracy.

We selected *Verticillium dahliae* as the experimental model. The choice of *V. dahliae* as a case study was based on the practical goal of our research: to help understand and control thuja wilt, of which *V. dahliae* is the main pathogen.

The proteome of *V. dahliae* is itself quite diverse. It encodes hundreds of secreted and effector proteins, including many carbohydrate-active enzymes and small cysteine-rich effector proteins involved in plant cell wall degradation.

The annotation coverage of dahlia virus is very high—essentially all predicted proteins have homologues in public databases, and a large proportion appear in curated resources. Together, these factors make the dahlia virus an ideal model for evaluating weighted distance matrix deep learning clustering and classification methods [21].

We employed AlphaFold2 to predict the tertiary structures of its proteins and compared these structures to elucidate the characteristics of proteins across different functional categories. Leveraging the structural information predicted by AlphaFold2 enabled us to achieve more precise protein classification, demonstrating higher efficiency and accuracy compared to traditional strategies that rely solely on 1D amino acid sequences. The sequence similarity within protein families was validated using BLAST sequence alignment.

We developed a novel deep learning UNSE model, based on the U-Net architecture and incorporating the Squeeze-and-Excitation attention mechanism, for clustering and classification of protein tertiary structures. We constructed distance matrices that consider only corresponding residue pairs based on the obtained E-values and alignment results. This approach ensures that the introduced sequence similarity information is accurately reflected in specific regions or residue combinations of the clustering matrix, thereby generating weighted distance matrices. Additionally, we employed data normalization, data augmentation, and PCA-based dimensionality reduction to ensure the model effectively captures the complex features inherent in protein structures. Furthermore, by integrating many algorithms, we achieved more detailed and multi-level classifications of proteins. Experimental results demonstrate that our method significantly outperforms traditional sequence-based BLAST and structure-based DeepGo methods in clustering accuracy across multiple datasets, particularly exhibiting enhanced adaptability and robustness when handling structurally complex proteins.

## Materials and methods

### Collection and preprocessing of baseline data set

Prior to testing, the framework we developed was first rigorously pretrained on a wider range of structural databases to obtain a more accurate feature extraction model (see Supplementary Data). We downloaded 10 M data from the Pfam database and performed data de-redundancy by 95% sequence identity to obtain 30 k nonredundant structural Protein Data Bank (PDB) data. At the same time, we also investigated the performance when incorporating SWISS-MODEL into training, which greatly increased the number of training samples and reduced the imbalance between positive and negative examples. In addition, 220 k nonredundant structured data from SWISS-MODEL were obtained through sequence homogeneity. In addition, we examined the cluster classification performance when using only PDB, only SWISS-MODEL, and both PDB and SWISS-MODEL and then divided the uniform nonredundant set into training, validation, and testing subsets in the ratio of 80%:10%:10%. Specific data have been uploaded in Supplementary Information Fig. 2. The parameters of the pretraining were fixed in the subsequent tests (see Supplementary Information Fig. 1).

The pretrained model was clustered and classified using the AlphaFold2-predicted protein tertiary structure of *V. dahliae* as a test dataset. AlphaFold2 accurately predicts tertiary structures from amino acid sequences [22] and is particularly well suited for handling sequences with low homology. Its efficiency and precision enhance the reliability of the data used in this research [23, 24]. The experimental design comprised three main components: To characterize the spatial geometric features of protein tertiary structures, Euclidean distances between Cα atoms in the PDB files were first calculated to construct structural distance matrices for each protein. The Cα atoms, being critical residues in the protein backbone, effectively reflect the core structure of proteins [24]. By computing the Euclidean distances between all pairs of Cα atoms, the spatial conformations of the proteins were precisely captured. Additionally, incorporating InterPro Entry (IPR) numbers from Pfam annotations, the distance matrices were weighted to emphasize functionally relevant structural features. By comparing the clustering results obtained from pure structural distance matrices with those derived from weighted distance matrices and contrasting them with sequence-based clustering methods, the effectiveness and biological significance of the clustering approach were further evaluated. Finally, the clustering results were validated, ensuring that the classifications are not only mathematically sound but also exhibit high consistency with biological functions.

This comprehensive data set processing approach aims to elucidate the spatial features of protein tertiary structures and their potential relationships with biological functions, thereby providing a robust foundation for accurate protein clustering and classification. To characterize the spatial geometric features of protein tertiary structures, the Euclidean distance between the Cα atoms of two residues *i* and *j* is calculated using the following formula:


(1) \begin{equation*} {d}_{ij}=\sqrt{{\left({x}_i-{x}_j\right)}^2+{\left({y}_j-{y}_j\right)}^2+{\left({z}_i-{z}_j\right)}^2} \end{equation*}

where ${d}_{ij}$ denotes the distance between the ith and the jth Cα atoms; ${x}_i$,${y}_i$, and ${z}_i$ are the 3D spatial coordinates of atom *i*, and ${x}_j$,${y}_j$, and ${z}_j$ are the 3D spatial coordinates of atom *j*; 𝑑𝑖𝑗 quantifies the straight-line distance between two 3D spatial atoms, thus reflecting both local and global folding characteristics.

All distance matrices were normalized to a 256 × 256 dimension in order to guarantee a uniform input format for protein structures of various sizes. Following the calculation of pairwise Euclidean distances for all Cα atom pairs within a protein, the resulting distance matrix is normalized to a [0, 1] range using the min-max normalization method:


(2) \begin{equation*} {\mathrm{x}}^{\prime }=\frac{x-\min (x)}{\max (x)-\min (x)} \end{equation*}

where x is the original data, x′ is the normalized data, and min(*x*) and max(*x*) are the minimum and maximum values in the distance matrix, respectively. This normalization ensures that all distance values are within a consistent range, enhancing the stability and performance of subsequent clustering algorithms.

To ensure consistency in the input format, the matrix size was adjusted using the bilinear interpolation method. This technique estimates the value at a noninteger position within a matrix by considering the four nearest integer positions and performing linear interpolation first in one direction (e.g. *x*-direction) and then in the other direction (e.g. *y*-direction). The bilinear interpolation formula is as follows:


(3) \begin{align*} \mathrm{I}\left(\mathrm{x},\mathrm{y}\right)=&\ \left(1-\mathrm{a}\right)\left(1-\mathrm{b}\right)\mathrm{I}\left(\mathrm{i},\mathrm{j}\right)+\mathrm{a}\left(1-\mathrm{b}\right)\mathrm{I}\left(\mathrm{i}+1,\mathrm{j}\right)\nonumber \\ &+\left(1-\mathrm{a}\right)\mathrm{bI}\left(\mathrm{i},\mathrm{j}+1\right)+\mathrm{a}\mathrm{bI}\left(\mathrm{i}+1,\mathrm{j}+1\right) \end{align*}

where: $\mathrm{I}\left(\mathrm{x},\mathrm{y}\right)$ is the interpolated value at the noninteger position (x, y). $\mathrm{I}\left(\mathrm{i},\mathrm{j}\right)$, $\mathrm{I}\left(\mathrm{i}+1,\mathrm{j}\right)$, $\mathrm{I}\left(\mathrm{i},\mathrm{j}+1\right)$, and $\mathrm{I}\left(\mathrm{i}+1,\mathrm{j}+1\right)$ are the values at the four nearest integer positions surrounding (x, y). a = x − ⌊x⌋ and b = y − ⌊y⌋ are the fractional parts of the coordinates (x, y), where ⌊x⌋ and ⌊y⌋ denote the integer parts of x and y, respectively.

This method ensures that the interpolated values smoothly transition between known data points, preserving the continuity and structure of the original matrix.

In order to enrich the dataset and improve the generalization ability of the model, this study employs data enhancement techniques including random affine transformations and random horizontal flipping. When working with complicated structural data, these modifications improve the model’s robustness and adaptability by simulating potential changes in protein conformation.

### Corresponding residue levels of protein family similarity

To ensure consistent amino acid correspondence when comparing distance matrices from different structures, a linkage between sequence and structural positions is established by aligning PDB sequences with Fast All (FASTA) sequences using Bio.PDB.PPBuilder. For handling multiple chains or models, a flattening method is applied to all models, chains, and residues, with a checking mechanism implemented to address discrepancies in Cα atom numbers between PDB files.

To improve data integrity, metadata such as PDB file length, sequence information, residue numbers, and the dimensions of distance matrices are stored separately. This facilitates subsequent verification of data completeness and comparability. Additionally, space is reserved for further dimensionality reduction and comparison methods, such as PCA, along with information on the explained variance of the principal components.

At the residue level, peptide sequences are extracted from PDB files using Biopython’s PDBuilder, followed by multiple sequence alignment (MSA) using BLAST. The alignment results are mapped to corresponding PDB residues, ensuring structural distances in the distance matrix align with sequence similarity data from BLAST. When residue-level correspondence is not possible, exact alignment of PDB and BLAST sequences is ensured prior to full-length comparison. Instead of uniformly scaling the entire distance matrix, each residue pair is weighted based on sequence alignment. Highly conserved regions identified by BLAST can either have reduced structural differences in the distance matrix or be highlighted by adjusting their weight accordingly.

The sequence similarity scores are first normalized, converting E-values or sequence identity into a similarity score ranging from 0 to 1. For each pair of residues (i, j) in the distance matrix, the corresponding sequence similarity score (s_ij) is applied to the following formula:


(4) \begin{equation*} {\mathrm{final}}_{\mathrm{distance}\left[\mathrm{i},\mathrm{j}\right]}={\mathrm{distance}}_{\mathrm{matrix}\left[\mathrm{i},\mathrm{j}\right]}\ast \mathrm{f}\left({\mathrm{s}}_{\mathrm{ij}}\right) \end{equation*}

where $\mathrm{f}\left(\mathrm{s}\_\mathrm{ij}\right)$ can be a function such as $\mathrm{f}\left(\mathrm{s}\_\mathrm{ij}\right)=1/\left(1+\mathrm{s}\_\mathrm{ij}\right)$ or $\mathrm{f}\left(\mathrm{s}\_\mathrm{ij}\right)=\left(1-\mathrm{s}\_\mathrm{ij}\right)$. This ensures that sequence similarity influences structural distances, making the matrix biologically meaningful. Additionally, E-values are transformed into similarity scores using a function such as $\mathrm{similarity}=-\log 10(\mathrm{E}-\mathrm{value} +1\mathrm{e}-10)$, which is then normalized. Residue-level weighting is applied to the distance matrix based on sequence alignment results, emphasizing conserved regions (with higher similarity corresponding to greater weight), thereby ensuring that the matrix reflects evolutionary relationships.

### Neural network feature extraction

In essence, U-Net’s hallmark skip connections enable the seamless fusion of high-resolution, local spatial characteristics—crucial for preserving the fine-grained topology of Euclidean distance matrices—with the deeper, more abstracted features learned by the encoder. This multi-scale feature integration ensures that subtle, residue-level interactions are not lost during the encoding process, thereby allowing the model to capture both local and global structural motifs. By contrast, architectures lacking such direct pathways (e.g. standard ResNet or pure Transformer models without explicit skip links) may fail to retain these detailed spatial patterns, which are essential for accurate protein structure representation.

The present investigation employed an improved U-Net autoencoder neural network model to augment the precision and efficiency of protein clustering classification [25, 26]. On this basis, we introduced the Squeeze-and-Excitation (SE) module into the U-Net architecture to enhance the model’s ability to model the dependency between feature channels. The SE module enhances the feature representation of the model by adaptively adjusting the channel weights to highlight the key features relevant to the clustering task [27–29].

The encoder component uses four convolutional layers to extract high-level features one layer at a time. Convolution with stride = 2 is used to do Downsampling. An SE module is introduced to capture the dependencies between global feature channels after each encoder layer. For the purpose of preserving multi-scale feature information, the decoder portion is up-sampled by transposing the convolutional layers, and the matching encoder layer features are fused using jump-joins. The final output of the model is generated by convolution and Sigmoid activation functions.

**Figure 1:**
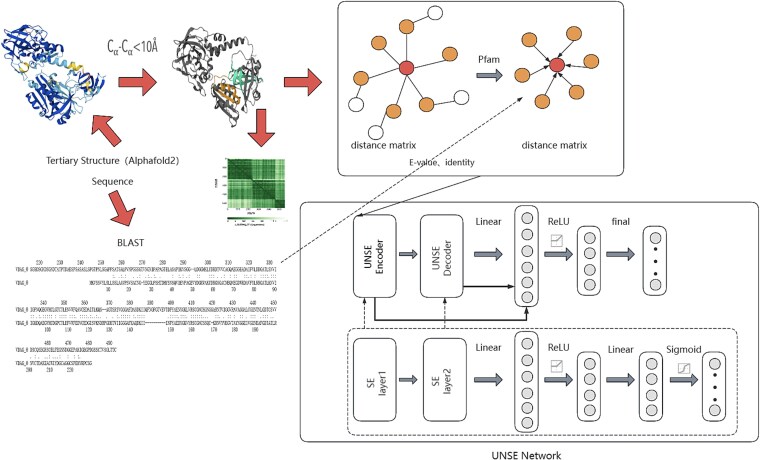
Schematic structure of the UNSE self-encoder model.

The introduction of the SE module empowers the model with a self-attention mechanism, which more effectively captures the non-linear relationships between features. The schematic diagram of the neural network structure is shown in Fig. 1.

Our improved U-Net autoencoder model leverages deep learning to automatically learn high-level feature representations and introduces the SE module, which further enhances the ability to model interchannel dependencies of features.

### Design of clustering classification algorithm

We chose a variety of classical clustering algorithms to analyze the data [30]. These include K-Means clustering, Gaussian Mixture Model (GMM), Agglomerative Hierarchical Clustering (Agg), and Density-Based Clustering Algorithm. Each of these algorithms may depict the data’s clustering structure from a different angle and have unique benefits.

K-Means clustering is a division-based clustering method whose objective is to divide n samples into K clusters such that the sum of squared errors of the samples within the clusters is minimized. $\left\{{x}_1,{x}_2,\dots, {x}_n\right\}\in{R}^d$ into K clusters ${C}_1,\dots, {C}_k$.

Its objective function is defined as:


(5) \begin{equation*} \operatorname{MIN}:\mathrm{J}={\sum}_{k=1}^K{\sum}_{x_i\in{C}_k}{\left\Vert{x}_i-{u}_k\right\Vert}^2 \end{equation*}

where ${C}_k$ denotes the set of samples of the *k*th cluster, ${u}_k$ is the centre of mass of the *k*th cluster, and $\left\Vert \cdotp \right\Vert$ denotes the Euclidean parameter. To increase the stability and clustering quality of the algorithm, we make multiple random initializations of the centre of mass position and select the clustering result that minimizes the objective function J.

The GMM is a probability density model that assumes that the data consist of a weighted mixture of K multivariate Gaussian distributions. Its probability density function is


(6) \begin{equation*} p(x)=\sum_{k=1}^K{\pi}_k\cdotp \mathrm{\aleph}\left(x|{\mu}_k,{\varSigma}_k\right) \end{equation*}

where ${\pi}_k$ is the mixing coefficient of the *k*th Gaussian component satisfying $\sum_{k=1}^K{\pi}_k=1$*;*  $\aleph \left(x|{\mu}_k,{\varSigma}_k\right)$ denotes the probability density function of the

$k$ -th Gaussian component, with mean ${\mu}_k$ and covariance matrix ${\varSigma}_k$. The parameters of the GMM model are estimated by the Expectation–Maximization (EM) algorithm to maximize the log-likelihood function of the observed data:


(7) \begin{equation*} \mathcal{L}=\sum_{i=1}^n\ln p\left({x}_i\right) \end{equation*}

${x}_i$ denotes the 𝑖th observation sample, which is a specific data vector (1D or multi-dimensional) observed from the dataset *D*.

$p\left({x}_i\right)$ then denotes the probability density of this sample point 𝑥𝑖 under the current parameters of the model, i.e. the value of the GMM fit to this point.

Agg is a bottom–up hierarchical clustering method in which each sample is initially treated as an independent cluster, and then, the most similar clusters are gradually merged until the stopping condition is satisfied. The distance between clusters can be calculated using various strategies such as single chain (shortest distance), full chain (longest distance), and average chain (average distance). Taking the average chain as an example, the cluster ${\complement}_i$ and ${\complement}_{k.}$ The distance between is defined as


(8) \begin{equation*} d\left({\complement}_i,{\complement}_k\right)=\frac{1}{\left|{\complement}_i\right|\cdotp \left|{\complement}_k\right|}\sum_{x_p\epsilon{\complement}_i}\sum_{x_q\epsilon{\complement}_k}\left\Vert{x}_p-{x}_q\right\Vert \end{equation*}

The $\left|{\complement}_i\right|$ and $\left|{\complement}_k\right|$ are the sample sizes of the clusters, respectively. ∥ ·∥ Default refers to the Euclidean parameter. ${x}_p$ denotes the 𝑝th data object in cluster ${\complement}_i$, an observation vector in the original dataset $D$. The outer summation $\sum{x}_p\epsilon\ {\complement}_i$, i.e. iterates over all the samples in cluster ${\complement}_i$. Similarly, ${x}_q$ denotes the *p*th data object in cluster ${\complement}_k$. The inner summation $\sum{x}_q\epsilon\ {\complement}_k$ traverses all samples in cluster ${\complement}_k$.

This formulation ensures that each pairwise distance contributes equally to the overall cluster distance.

The density-based clustering algorithm (DBSCAN) discovers arbitrarily shaped clusters by identifying high-density regions and is able to efficiently deal with data containing noise. For a given neighbourhood radius $\varepsilon$ and minimum number of points MinPts, a point ${x}_i\in D$ is classified as a core point if:


(9) \begin{equation*} \left\{{x}_i\ \left|\ \right|\ \left\{{x}_j\in D\ |\ \left\Vert{x}_i-{x}_j\right\Vert \le \varepsilon \right\}\ge \mathrm{MinPts}\right\} \end{equation*}

where $D$ denotes the dataset and ∥·∥ the Euclidean norm. Points that are density-reachable from a core point—i.e. connected by a chain of core points each within ε of the next—belong to the same cluster. All remaining points that are neither core points nor density-reachable are treated as noise. The algorithm terminates once all points in D have been assigned to a cluster or designated as noise.

### Model training and parameterization

Throughout the neural network model’s training phase, we employ an early stopping technique strategy to reduce overfitting and enhance the model’s capacity for generalization. At the conclusion of every training cycle, the validation set’s loss function value is specifically tracked. The training procedure is stopped early when the validation loss either stops decreasing or exhibits an upward trend for a certain number of cycles (preset patience values) [31]. This strategy effectively prevents the model from overfitting noise and specific patterns in the training data and ensures robust model performance on unknown data [32].

The high-dimensional deep features can be reduced to the properties of low-dimensional space using PCA [33], which maximally preserves the main variance information of the data. PCA deals with the following optimization issues:


(10) \begin{equation*} \operatorname{MAX}:\mathrm{Tr}\left({\mathrm{W}}^{\mathrm{T}}\mathrm{SW}\right),{\mathrm{W}}^{\mathrm{T}}\mathrm{W}=\mathrm{I} \end{equation*}

where $\mathrm{S}$ is the covariance matrix of the data, $\mathrm{W}$ is the projection matrix, and $\mathrm{Tr}\left(\cdotp \right)$ denotes the trace of the matrix. By selecting the eigenvector corresponding to the largest eigenvalue, PCA projects the data into a subspace consisting of principal components.

To ensure equitable weight allocation across BLAST E-values, Pfam, and Gene Ontology (GO) annotations, our methodology implements a three-stage harmonization process:

BLAST E-values were subjected to a negative logarithmic transformation (−log₁₀) to convert exponentially distributed E-values into linearly interpretable confidence scores. This transformation effectively mitigates the dominance of extreme E-value magnitudes while preserving their relative probabilistic significance.

Pfam and GO annotations are encoded as binary indicators (presence = 1, absence = 0) to capture discrete functional annotations without imposing arbitrary quantitative biases.

All transformed features were then unified through min-max normalization (scaled to the [0,1] range) to eliminate scale discrepancies between continuous E-values and categorical annotations. Crucially, we further incorporated Shannon information content—calculated from residue conservation profiles—as an adaptive weighting factor during model training. This step preferentially amplifies features with high biological specificity while suppressing noisy or ubiquitous structural patterns. The combined approach ensures that both statistical robustness (via scale normalization) and biological relevance (via information-theoretic weighting) are systematically embedded within the weighting matrix. We acknowledge that alternative normalization schemes could be explored in future work but emphasize that our current strategy draws upon well-validated precedents in multi-omics data integration.

While AlphaFold2’s predicted structures may exhibit local inaccuracies (e.g. flexible loop regions), its global fold prediction reliability—particularly for core Cα atom placements—has been extensively validated through independent assessments. As demonstrated in the CASP14 benchmark [23], AlphaFold2 achieves a median backbone RMSD of 1.0 Å across high-confidence predictions (pLDDT >90), with errors predominantly localized to surface-exposed residues rather than structurally conserved cores [34]. Our methodology inherently mitigates such localized errors through two key design choices:

- Distance matrix focus: By prioritizing pairwise Cα-Cα Euclidean distances over absolute coordinate precision, our approach emphasizes relative spatial relationships that remain stable under global structural alignments. This confers robustness to minor backbone deviations (e.g. RMSD <2.5 Å).
- Conserved residue pair weighting: The integration of Shannon entropy–based conservation scores preferentially weights evolutionarily constrained residue pairs, which typically reside in structurally stable functional regions less prone to prediction errors. This biological effectively filters out noisy distances from low-confidence regions.

Crucially, our approach constructs distance matrices solely from the relative positions of Cα atoms, meaning that such small local deviations fall well within the noise tolerance of the Euclidean distance topology.

As a result, the method is inherently robust to residual prediction errors: even if minor RMSD fluctuations occur, the overall spatial relationships that underpin our feature representations remain stable, ensuring reliable downstream analysis.

In this study, all hyperparameters are determined by grid search based on the performance of the model on the validation set. We randomly selected ~10% of the samples from the training set to form the validation set. To avoid overfitting, we applied an early stopping criterion of PATIENCE = 5 to the model (i.e. we stopped training if the validation loss did not improve within five cycles). We also used the ADAM optimizer with learning rates lr = 0.0001, β1 = 0.95 and β2 = 0.95, a batch size of 64, and a default number of cycles of 200. In addition, UNSE was implemented to handle variable-length sequences by performing sequence/contact map filling.

## Results

### Model training procedure and automated learning rate scheduling

**Figure 2:**
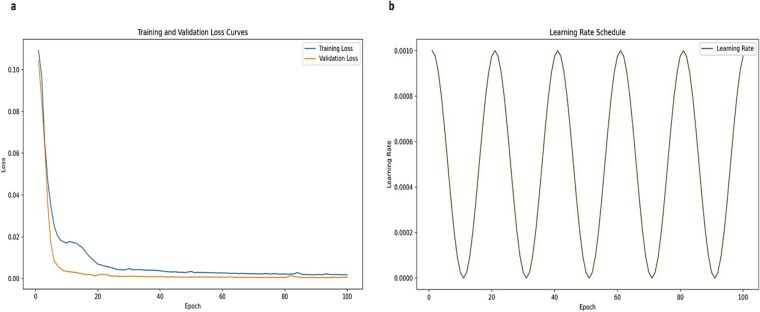
Loss value and learning rate change chart.

During the model training process, we recorded the changes in the loss function values of the training and validation sets with the number of iterations (shown in Fig. 2). As the number of training rounds increases, both the training loss and the validation loss show a decreasing trend and stabilize at about the 50th cycle, which indicates that the model successfully learns the deep features of the protein data without overfitting. To prevent overfitting, we adopted an early stopping strategy to terminate the training process early when the validation loss no longer decreases in 10 consecutive cycles. Eventually, the model achieved an optimal loss value of 0.0012 on the validation set, showing excellent generalization ability.

Throughout training, this model’s learning rate is dynamically altered. Using the Cosine Annealing Scheduler technique, the starting learning rate is set to 0.001 and is progressively decreased over 100 training cycles. This strategy accelerates the convergence speed of the model at the beginning of training and then achieves fine-tuning of the parameters by decreasing the learning rate at a later stage, which improves the stability and final performance of the model. The instability brought on by the high learning rate during the training phase is successfully avoided by the steady decrease in the learning rate. As illustrated in Fig. 2, this guarantees that the model can have superior generalization capabilities at a reduced rate of learning.

### Deep feature extraction and dimension reduction

In the feature extraction process, the protein distance matrix is input into a network consisting of an encoder and SE module. Each convolutional layer applies a stride of 2, progressively reducing the feature map size while increasing the number of channels to capture richer features. The SE module extracts global spatial information via global average pooling and recalibrates the channel responses through a fully connected layer and activation function.

After encoding, we obtain high-dimensional (256 × 256) features, which are flattened into 1D vectors to reduce computational complexity. However, high-dimensional data can lead to ‘dimensionality catastrophe’, which hinders clustering. To address this, we apply PCA to reduce the dimensionality and preserve the variance of the data.

**Figure 3:**
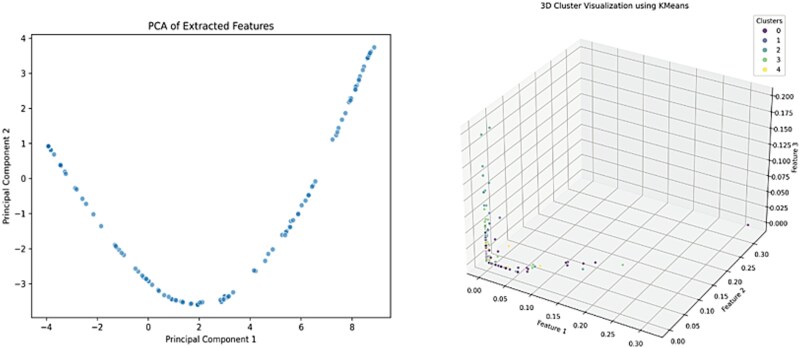
Distribution of reduced depth features in space.

In this study, we perform dimensionality reduction by projecting the high-dimensional features onto a low-dimensional space, simplifying analysis and visualization. The covariance matrix of the features is computed, and the two principal eigenvectors are selected to form a new feature space. The combined use of the improved UNSE encoder and PCA effectively retains key information while enhancing clustering performance. The resulting 2D features demonstrate excellent clustering performance and are easily visualized, confirming the method’s effectiveness. The feature distribution post-reduction, shown in Fig. 3, reveals distinct clustering regions, indicating the potential clustering structure.

### Clustering results with weighted structure

To assess the impact of neural network feature extraction on the effectiveness of protein clustering and classification, we conducted a comparative analysis of clustering outcomes based on the original distance matrix (without feature extraction) and the weighted distance matrix (incorporating Pfam annotation IPR numbers). The original distance matrix was directly derived from calculating the Euclidean distances between Cα atoms, serving as a baseline for traditional clustering methods. In contrast, the weighted distance matrix utilized features extracted by our proposed deep learning model to apply weights to the original distances, thereby emphasizing functionally relevant structural characteristics.

**Figure 4:**
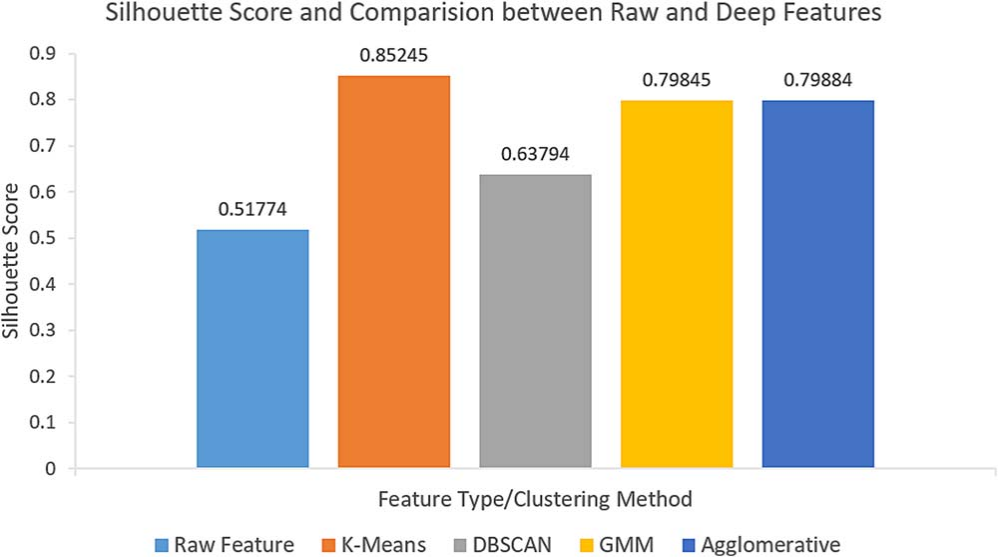
Comparison of contour coefficients of clustering results for raw and deep features.

In our experiments, we employed K-Means, GMMs, and Agg algorithms to analyze both types of distance matrices. Clustering performance was evaluated using the Silhouette Score metric. The results demonstrated that clustering methods based on the weighted distance matrix significantly outperformed those based on the original distance matrix across all evaluation metrics (as illustrated in Fig. 4). Additionally, the clustering results derived from the weighted distance matrix exhibited a high degree of consistency with the known biological functions in the Pfam database. The biological relevance of the clustering was further validated through BLAST sequence alignment and Gene Ontology (GO) annotations (refer to the Supplementary Data).

These findings indicate that the clustering approach, which integrates deep learning feature extraction with functional weighting, not only excels mathematically but also benefits from the enhanced discriminative power and effectiveness of the neural network–extracted deep features. Furthermore, it significantly improves the ability to elucidate the true biological functions of proteins, thereby validating the efficacy and practical applicability of our method.

**Figure 5:**
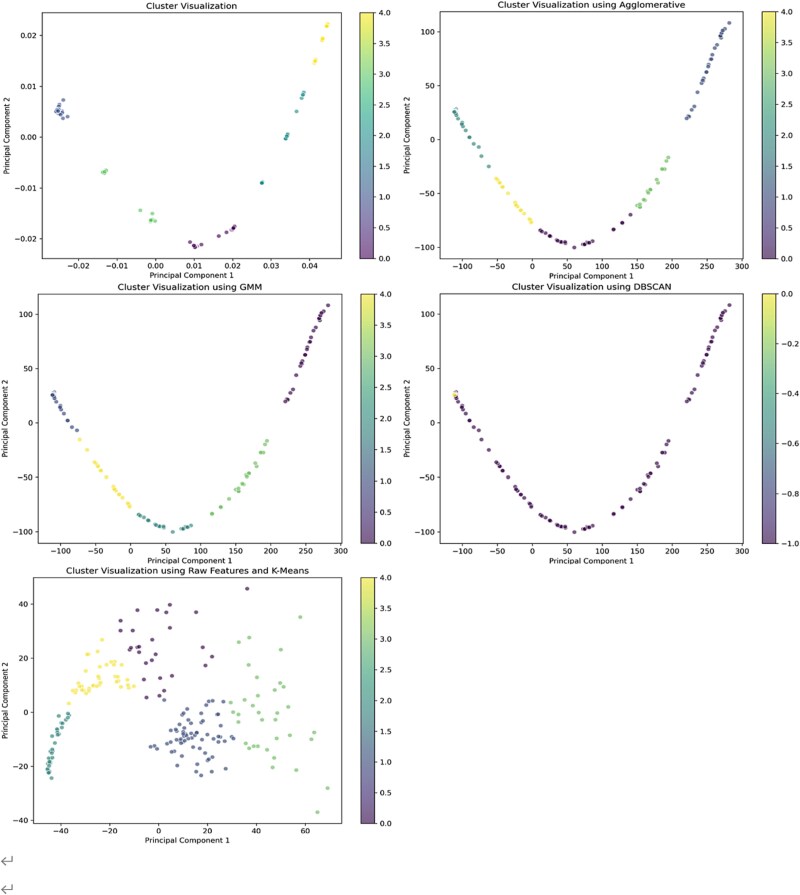
Visualization of clustering results in feature space.

The results show that the clustering results have a high degree of tightness and separation. The visualization of the clustering results is shown in Fig. 5, which demonstrates that the samples have been effectively divided into five clusters.

### Comparison with other state-of-the-art methods

In this study, we employed ${F}_{max}$ and AUPR (Area Under the Precision–Recall Curve) as the primary evaluation metrics to assess the performance of our protein sequence clustering and classification method based on Pfam annotations (IPR numbers). ${F}_{max}$ integrates precision and recall, providing a balanced assessment of the clustering results in terms of both accuracy and completeness, thereby reflecting the method’s ability to capture protein functional domains. The AUPR evaluates the model’s capability to distinguish true positives from false positives across various thresholds, further validating the robustness and accuracy of the clustering outcomes.

**Figure 6:**
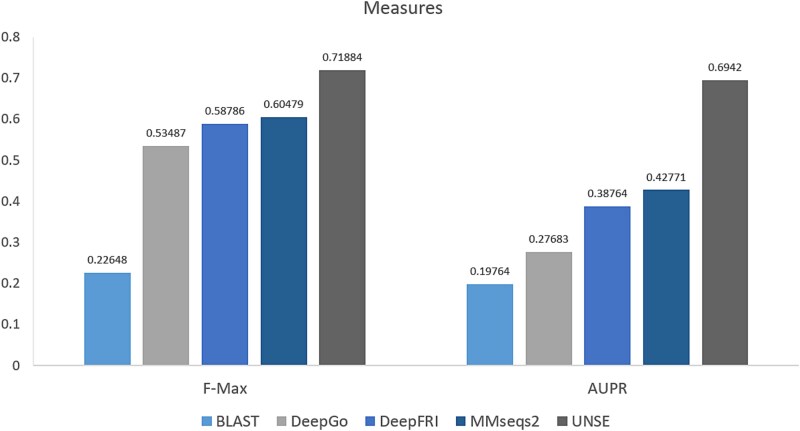
Comparison with other evaluation methods based on sequence and structure clustering.

During the evaluation process, we utilized a multi-label classification approach, selecting multiple IPR numbers to represent different functional domains. The results indicated that the generated distance matrices and clustering outcomes were highly consistent with the known biological functions in the Pfam database. Comparative experiments against sequence-based BLAST and other structure-based clustering classification methods (e.g. DeepGo, DeepFRI, MMseqs2) revealed that the high ${F}_{max}$ and AUPR scores demonstrate that our clustering approach not only performs well mathematically but also exhibits significant consistency in recognizing biological functions. This outcome validates the effectiveness of our clustering method in reflecting true biological correlations (shown in Fig. 6).

In addition to this, to explore the performance of our approach on different structural prediction tools, we conducted high-tolerance test experiments on model clustering classification performance. The results show that our model has a good tolerance under different qualities of data. For details, see Supplementary Information Fig. 3.

### Analysis of biological significance of clustering results

Cluster analysis of the tertiary structure of *V. dahliae* proteins revealed significant functional heterogeneity and specificity of proteins of this pathogenic fungus (as illustrated in Table 1).

**Table 1:** Deep clustering classification results (refer to the supplementary data).

**Clustering**	**Make up**
Ligase activity clustering (clustering 0)	VDAG_00107 VDAG_00261 VDAG_01417 VDAG_02034 VDAG_02751 VDAG_02770 VDAG_02891 VDAG_02908 VDAG_03321 ……
ATP binding function clustering (clustering 1)	VDAG_00063 VDAG_00166 VDAG_00274 VDAG_00482 VDAG_00493 VDAG_00600 VDAG_00684 VDAG_00752 VDAG_00900 ……
Protein function binding clustering (clustering 2)	VDAG_00076 VDAG_00481 VDAG_00559 VDAG_01039 VDAG_01839 VDAG_03128 VDAG_03221 VDAG_03375 VDAG_03377 ……
Clustering of transferase activities (clustering 3)	VDAG_00191 VDAG_00621 VDAG_01959 VDAG_02042 VDAG_02835 VDAG_03414 VDAG_03513 VDAG_03790 VDAG_05042 ……
Clustering of hydrolytic enzyme activities (clustering 4)	VDAG_01231 VDAG_01291 VDAG_01586 VDAG_01595 VDAG_02122 VDAG_02273 VDAG_02467 VDAG_02501 VDAG_02669 ……

Through in-depth analysis of the molecular functions of the clustered proteins, the study reveals the differentiation of *V. dahliae* proteins in terms of their biological functions. Adenosine Triphosphate (ATP) binding in Cluster 1 [34], protein binding in Cluster 2 [35, 36], ligase activity in Cluster 0 [23, 37], transferase activity in Cluster 3 [38, 39], and hydrolase activity in Cluster 4 [39, 40] collectively outline a complex network of functions in the signal transduction [41], gene expression regulation, metabolic homeostasis, and pathogenicity of *V. dahliae’s* complex functional network in biological processes such as signalling, gene expression regulation, protein modification, metabolic homeostasis, and pathogenicity. These findings are not only consistent with the existing literature on the pathogenic mechanism of *V. dahliae* but also further deepen the understanding of its pathogenic potential and environmental adaptation strategies.

### Proof of protein family similarity

To validate the protein family similarities within the *V. dahliae* protein family, we performed sequence alignments using BLAST, with the results summarized in Table 2. The sequence identity percentages consistently exceeded 70%, indicating a high degree of conservation within the *V. dahliae* family. Additionally, the obtained e-values were extremely low (e.g. <1e-15), confirming the statistical significance of these similarities. The MSA presented in the attachment highlights conserved regions that play critical roles in maintaining the structural integrity and functional specificity of the *V. dahliae* proteins. These conserved residues directly correspond to important positions within the distance matrices, providing substantial evidence of the correlation between sequence conservation and structural similarity.

**Table 2:** Partial BLAST sequence alignment results of VDAG_00063.

**Protein**	**Bits**	**E-value**	**%_Sim**	**Alen**
VDAG_03354	998	3.4e-30	77.7	296
VDAG_06155	707	1.3e-19	70.0	257
VDAG_07768	697	2.8e-19	70.5	224
VDAG_08685	583	5.2e-19	70.6	224

The alignment of MSA results onto structural models revealed that conserved regions are predominantly located within core structural domains, which are essential for protein stability and function. This correspondence between sequence-based similarity and structural distance metrics underscores the robustness of our deep learning-based clustering and classification methodology for *V. dahliae* proteins. By integrating BLAST sequence alignments with structural analysis, we capture not only the evolutionary relationships but also the functional correlations within the *V. dahliae* protein family. This validation of similarity enhances the reliability and biological relevance of our protein tertiary structure classification model. Detailed data can be found in the Supplementary data.

## Discussion

This study introduces a novel protein classification method that integrates deep learning with clustering algorithms, using an autoencoder model based on the U-Net architecture, enhanced by the SE attention mechanism. This approach effectively processes and clusters the tertiary structure protein data of *V. dahliae*. Compared to traditional clustering methods that rely solely on sequence information, our proposed model significantly improves the ability to capture intricate features of protein structures. It is particularly effective in classifying proteins that share similar functions but exhibit substantial sequence differences, which is a common challenge for conventional sequence-based approaches.

In this study, we selected *V. dahliae* and used AlphaFold2 to predict its protein structures [42], recognizing that while AlphaFold2 excels at predicting overall protein folding, it is less sensitive to point mutations. To address this, our focus is on clustering and classification based on the predicted structural folds, which are robustly captured by AlphaFold2. We extracted PDB files and calculated Euclidean distances between Cα atoms to generate initial distance matrices. These matrices underwent feature extraction followed by PCA, which retained over 90% of the variance in the data. To enhance the biological relevance of the clustering, we introduced weighted distance matrices, which integrate BLAST sequence alignment E-values, Pfam annotations, and GO annotations, focusing on conserved residue pairs that reflect functional regions of the proteins. Given the absence of a clear ‘gold standard’ for clustering validation, we employed biological data from GO annotations as an additional measure of biological significance, ensuring that the clusters correspond to known functional categories. This step bridges the gap between structural data and functional interpretation, providing a clearer understanding of the relationships between protein structures and their biological roles. We applied four machine learning clustering algorithms to both raw and weighted distance matrices, comparing their performance against results obtained without neural network feature extraction. Furthermore, we benchmarked our method against other structure-based clustering approaches, using GO annotations to validate the biological relevance of the clusters [43]. Experimental results demonstrated that clustering based on weighted distance matrices significantly outperforms raw distance matrices across key metrics, including Silhouette Score [44], ${F}_{max}$, and AUPR [42, 45], and surpasses traditional sequence-based and other structure-based clustering methods. These findings address the lack of a gold standard by validating clustering results with biological annotation data, offering a more accurate assessment of protein structure and function. By integrating deep learning with weighted distance matrices, this approach captures both structural and functional features, ensuring robustness and biological relevance in structural biology.

This improvement not only demonstrates the mathematical efficacy of deep learning feature extraction but also highlights the ability of the weighted distance matrices to align clustering outcomes with the functional properties of proteins. Such alignment is crucial for advancing functional genomics, drug discovery, and gene therapy, as it offers deeper insights into the structural basis of protein function, which can guide the development of more targeted therapeutic strategies.

However, there are several limitations in this study. First, although PCA was employed to reduce the dimensionality of high-dimensional features, enhancing clustering efficiency and interpretability, there is still a risk of information loss, despite retaining ~90% of the variance. The effectiveness of this dimensionality reduction strategy needs further validation when applied to larger and more complex datasets. Additionally, this study focused primarily on the protein dataset from *V. dahliae*. Extending the method to include other protein families will be essential to assess its generalizability. While AlphaFold2 excels in predicting protein structures, its sensitivity to point mutations is limited. To further improve the reliability and stability of the predictions, future research could incorporate experimental validation data or multi-model cross-validation to enhance prediction accuracy.

In conclusion, this research successfully integrates 3D structural information into a deep learning–based clustering model, overcoming the limitations of traditional sequence-based methods and significantly enhancing the accuracy and robustness of protein clustering and classification. The method not only demonstrates mathematical superiority but also reveals high consistency in identifying the true biological functions of proteins. By incorporating biological annotations and validating clustering results through experimental data, we ensure the biological relevance of the approach. Future studies will focus on optimizing algorithm efficiency, improving scalability for larger datasets, and addressing the method’s limitations, thereby advancing its application in structural biology, drug design, and gene editing.

**Key Points**

- The proposed UNSE autoencoder architecture effectively captures the 3D features of protein structures, offering an innovative solution to overcome the limitations of traditional sequence-based methods.
- Incorporating weighted distance matrices that integrate sequence-based annotations (such as GO and Pfam) into the clustering process bridges the gap between structural and functional data, ensuring that the clusters correspond to biologically meaningful categories, which enhances the biological relevance of the analysis.
- By focusing on clustering proteins based on AlphaFold2’s predicted structural folds rather than relying on sequence data, this method addresses the limitations of sequence-based approaches.

## Supplementary Material

supplementary_files_bbaf331

## Data Availability

The source code for the simulation and real data application is available at https://github.com/2935361139/protein_clustering/tree/master The PDB dataset used in this article is available at https://alphafold.ebi.ac.uk/. BLAST sequence alignment website https://blast.ncbi.nlm.nih.gov/Blast.cgi?PROGRAM=blastp&PAGE_TYPE=BlastSearch&LINK_LOC=blasthome. The website for Pfam function annotation is http://pfam.xfam.org/.
