# Supplementary material for: Deep learning–enhanced clustering and classification of protein molecule tertiary structures using weighted distance matrices
Source: Brief Bioinform. 2025 Jul 8;26(4):bbaf331. doi: 10.1093/bib/bbaf331 (PMC12234446; doi:10.1093/bib/bbaf331)
Supplement: supplementary_files_bbaf331 [file supplementary_files_bbaf331.zip › Supplementary_information_bbaf331.docx]

**Deep Learning-Enhanced Clustering and Classification of Protein Molecule Tertiary Structures Using Weighted Distance Matrices**

Junlong Liu ^1^ ,Jiaming Xiao^1^ , Xunwen Su^1,2*^ , Yonglin Wang^3*^

^1^ School of Technology, Beijing Forestry University, Beijing 100083, China.

^2^ National Facility Preservation Bank for Forestry and Grassland Germplasm Resources, Beijing Forestry University, Beijing 100083, China.

^3^ State Key Laboratory of Efficient Production of Forest Resources, College of Forestry, Beijing Forestry University, Beijing, 100083, China. College of Forestry, Beijing Forestry University, Beijing,100083, China.

Corresponding author: X. Su; Email: [suxw0703@gmail.com](mailto:suxw0703@gmail.com)

Y. Wang; Email: [ylwang@bjfu.edu.cn](mailto:ylwang@bjfu.edu.cn)

**Supplementary Note**

Pre-training structure (LSTM-LM)

Input: 1-hot encoding of sequence: (n_samples, L, 26).

- LSTM (dim = 512, return_sequences=True, kernel_constrain=MinMax(-2.0,2.0), recurrent_constrain=MinMax(-2.0, 2.0))
- LSTM (dim = 512, return_sequences=True, kernel_constrain=MinMax(-2.0,2.0), recurrent_constrain=MinMax(-2.0, 2.0))
- TimeDistributed(Dense(26))
- Activation (softmax)

Optimization: loss = categorical_crossentropy; optimizer = Adam (lr = 0.001, β1 = 0.99,β2= 0.99); batch_size = 128; epochs = 5.

**Supplementary Figure 1.** We trained the LSTM language model by extracting 10 M sequences from the complete set of sequences of Pfam. These sequences are represented using 1-hot coding. The language model architecture consists of two stacked forward LSTM layers with 512 cells each.The LSTM-LM model is trained with 5 calendar elements using the ADAM optimizer with a learning rate of 0.001 and a batch size of 128.All hyperparameters are determined by grid search based on the model's performance on the validation set.


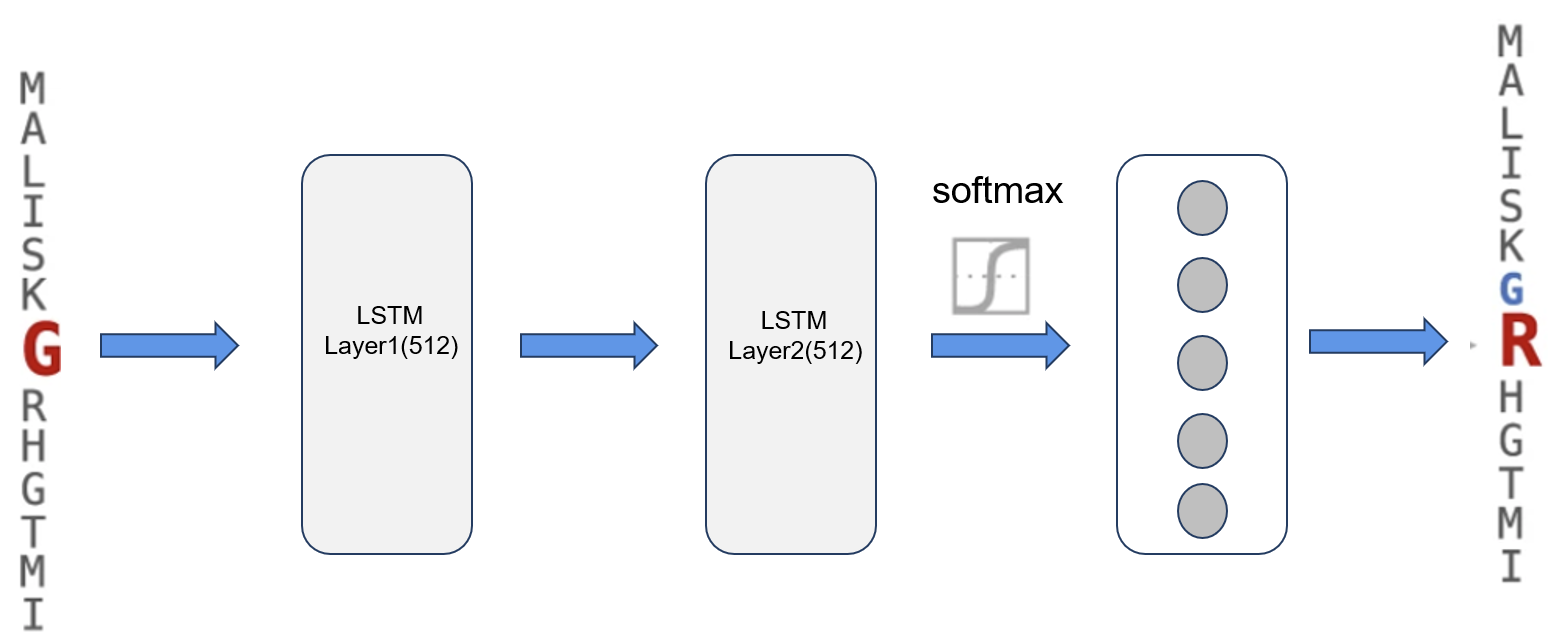


**Supplementary Figure 2.** We examined the performance of our method when trained on PDB only, on SWISS-MODEL only, and on both PDB and SWISS-MODEL. For example, Fig. a shows the effect of clustering and categorizing on PDB only, Fig. b shows the effect of clustering and categorizing on SWISS-MODEL only, and Fig. c shows the effect of clustering and categorizing on PDB and SWISS-MODEL at the same time.


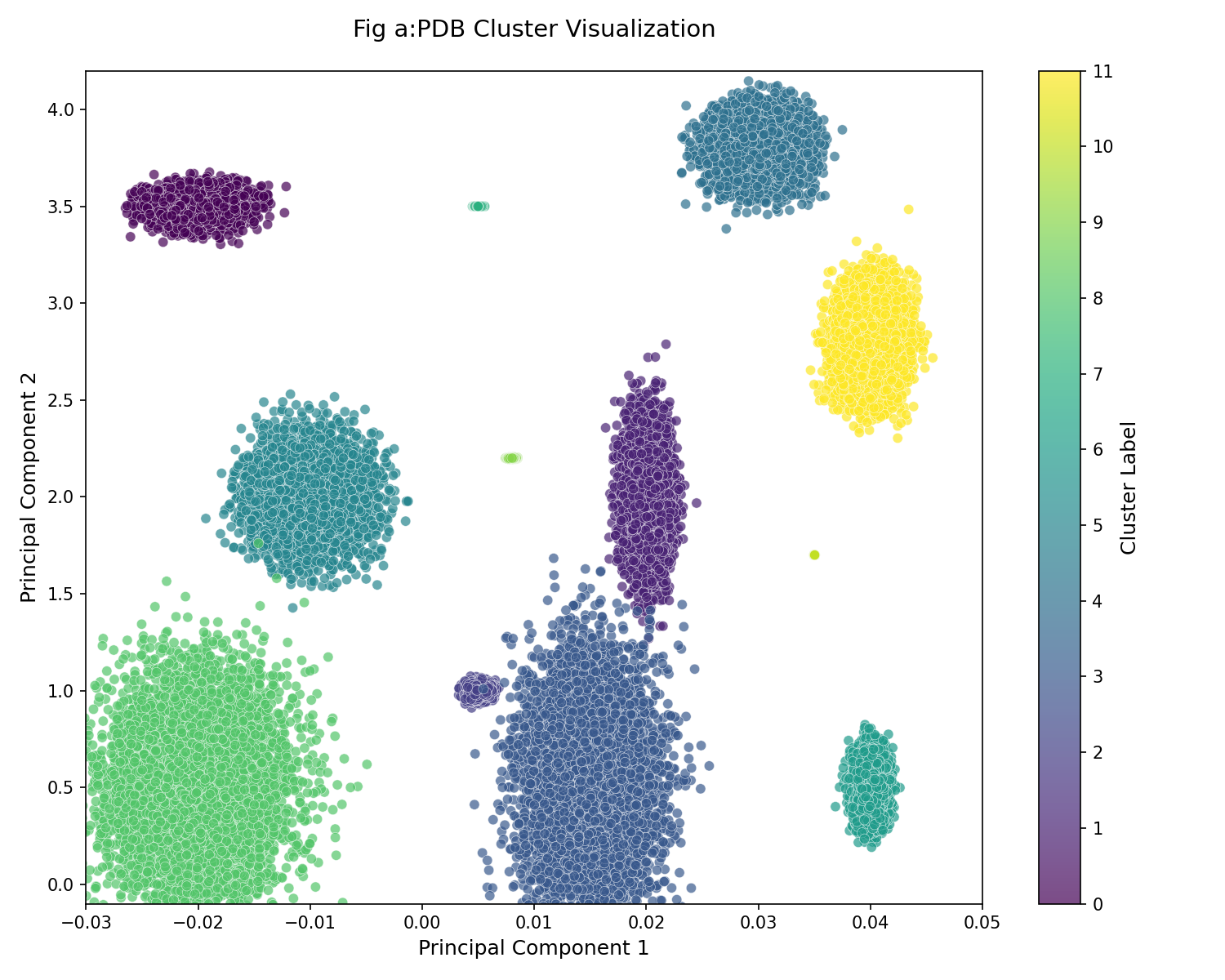

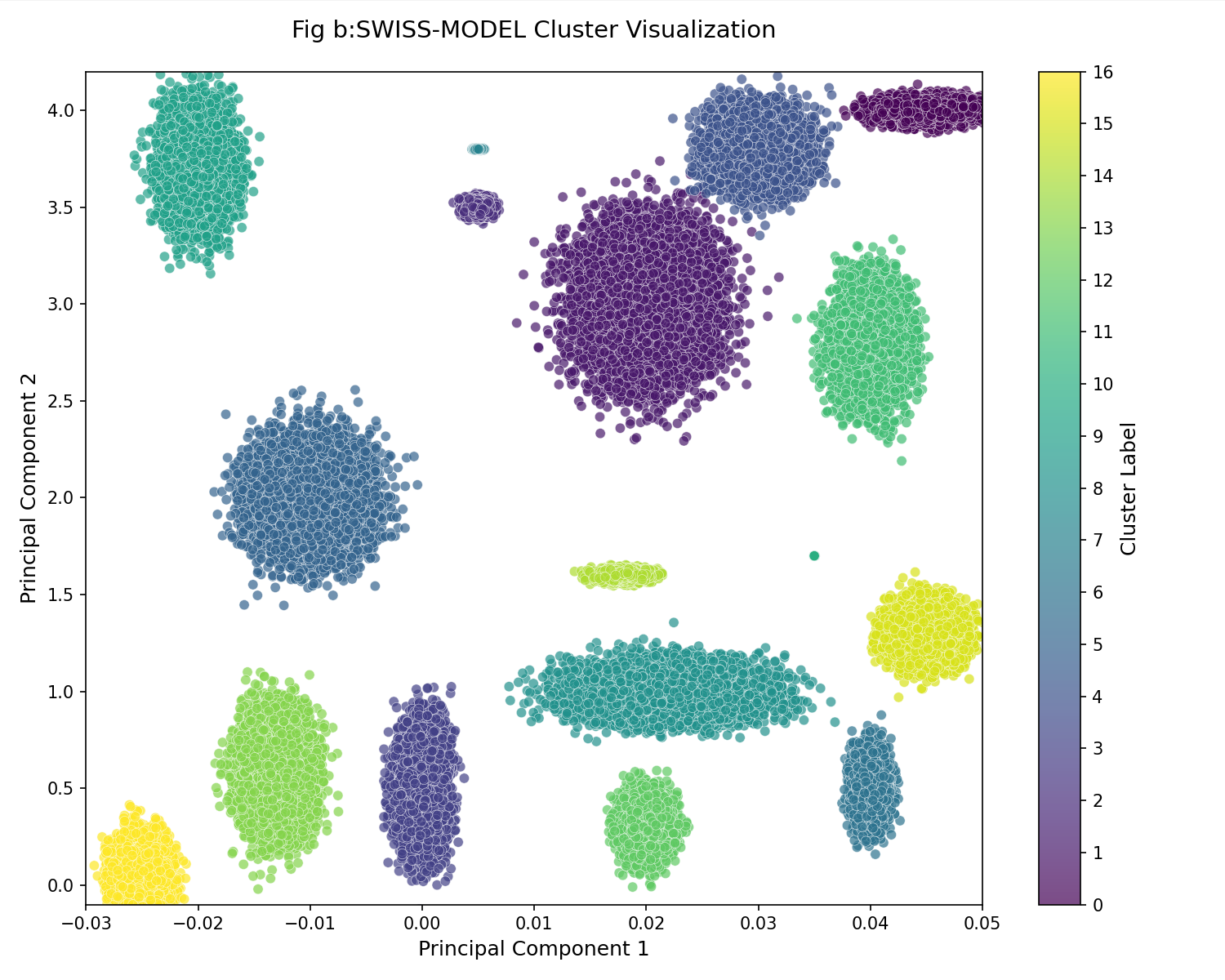

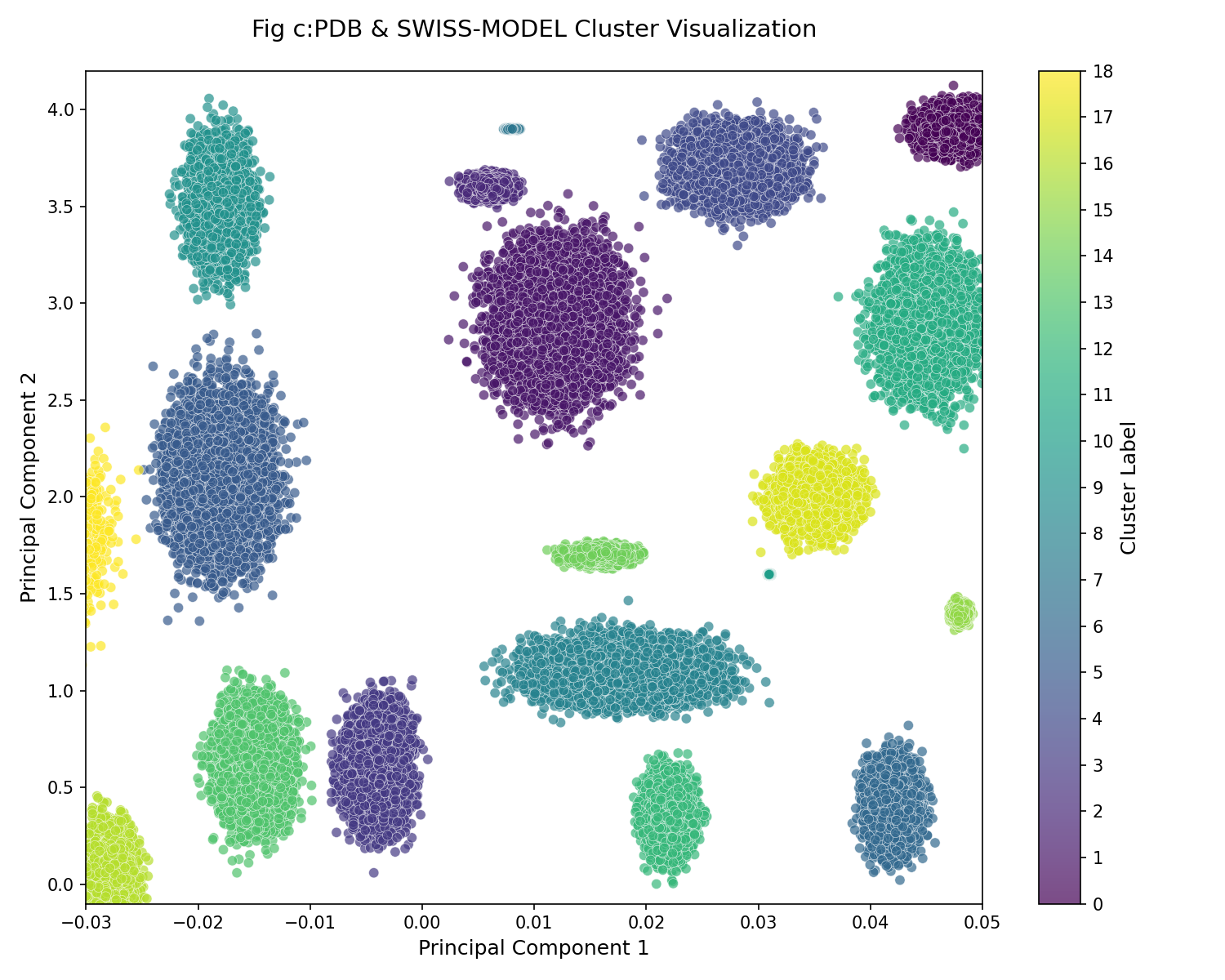


**Supplementary Figure 3.** The tertiary structure data tolerance for training UNSE on PDB structures was explored by comparing the performance of our method on structure data obtained from Alphafold2 and other structure prediction methods. The specific comparison steps were as follows: we input 765 sequences into each of the three methods, alphafold2, Rosetta, and DMPfold, for structure prediction, resulting in three different qualities of tertiary structure (the sequence data are shown as blue lines in Supplementary Fig. 3). This is because of the fact that among the known methods, alphafold2 predicts structures with the highest accuracy and best quality, whereas Rosetta and DMPfold predict structures of poorer quality and generate noise. In order to further test the robustness and fault tolerance of our method for cluster classification in predicting the degradation of structural data quality, we conducted cluster classification comparison experiments on data from different structural prediction methods. The experimental results show that the performance of our method on three different structure prediction tools is not significantly affected, which indicates that UNSE has a strong denoising capability. As shown in the figure, the data predicted by alphafold2, Rosetta, and DMPfold structural prediction tools can all reach F1 scores of 0.6 or more at a recall of 0.6, which shows the high denoising ability and high tolerance of our model, but the F1 scores of the sequence-based data cannot reach 0.6.


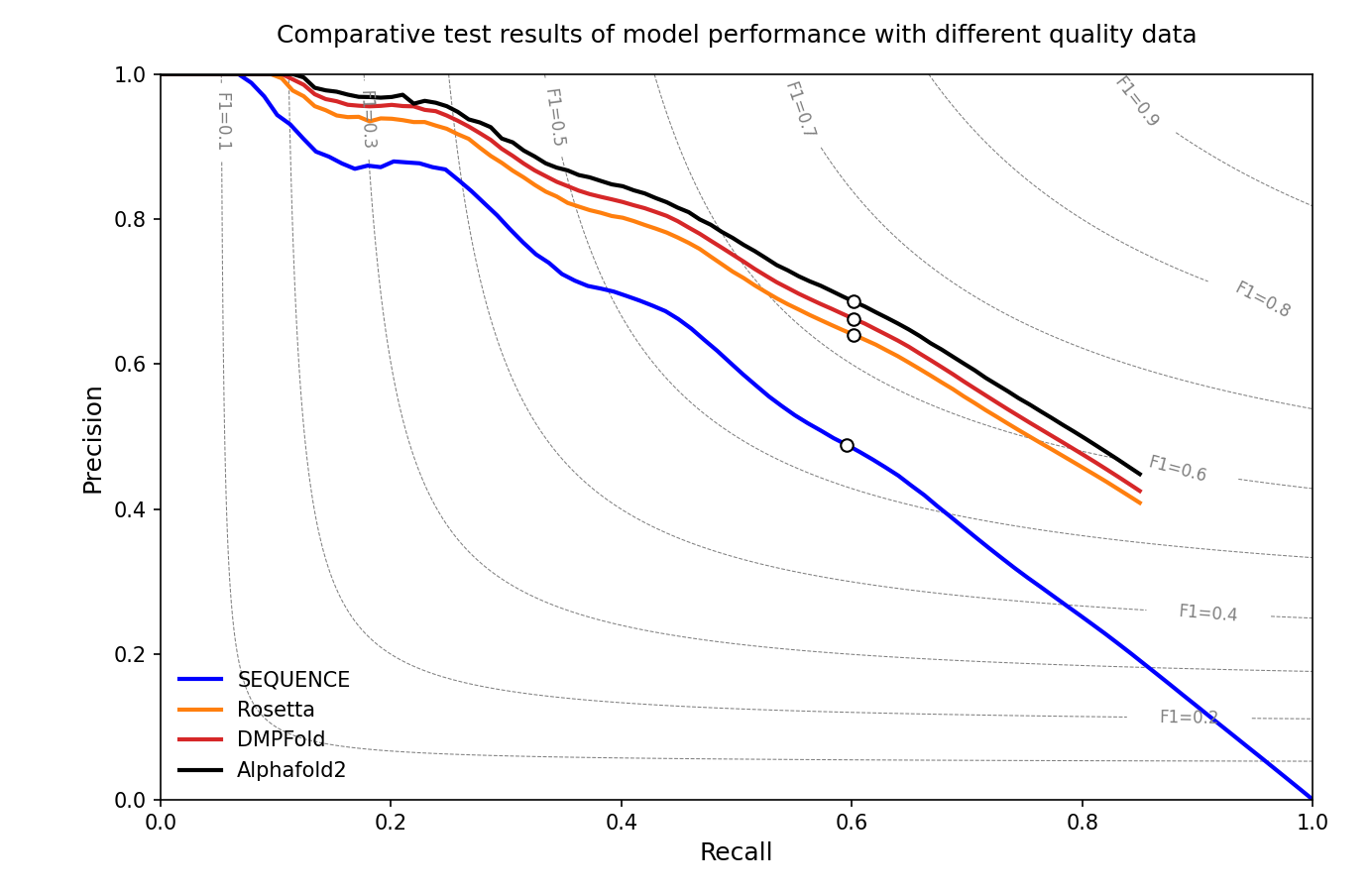


Command Line and Manual

**DeepGO-DeepFRI**

mkdir $DATA_DIR

printf "\n\n DATA DIRECTORY (%s) CREATED!\n" $DATA_DIR

printf "\n\n DOWNLOADING SIFTS-GO DATA...\n"

wget ftp://ftp.ebi.ac.uk/pub/databases/msd/sifts/flatfiles/tsv/pdb_chain_go.tsv.gz -O $DATA_DIR/pdb_chain_go.tsv.gz

printf "\n\n DOWNLOADING SIFTS-EC DATA...\n"

wget ftp://ftp.ebi.ac.uk/pub/databases/msd/sifts/flatfiles/tsv/pdb_chain_enzyme.tsv.gz -O $DATA_DIR/pdb_chain_enzyme.tsv.gz

printf "\n\n DOWNLOADING PDB SEQRES SEQUENCES...\n"

wget ftp://ftp.wwpdb.org/pub/pdb/derived_data/pdb_seqres.txt.gz -O $DATA_DIR/pdb_seqres.txt.gz

printf "\n\n DOWNLOADING PDB CLUSTERS...\n"

wget https://cdn.rcsb.org/resources/sequence/clusters/bc-$SEQ_SIM.out -O $DATA_DIR/bc-$SEQ_SIM.out

printf "\n\n DOWNLOADING GO HIERARCHY...\n"

wget http://purl.obolibrary.org/obo/go/go-basic.obo -O $DATA_DIR/go-basic.obo

printf "\n\n PREPROCESSING GO-ANNOTATIONS [Please wait this process may take a few minutes]...\n"

python create_nrPDB_GO_annot.py \

-sifts $DATA_DIR/pdb_chain_go.tsv.gz \

-bc $DATA_DIR/bc-$SEQ_SIM.out \

-seqres $DATA_DIR/pdb_seqres.txt.gz \

-obo $DATA_DIR/go-basic.obo \

-out $DATA_DIR/nrPDB-GO \

printf "\n\n PREPROCESSING EC-ANNOTATIONS [Please wait this process may take a few minutes]...\n"

python create_nrPDB_EC_annot.py \

-sifts $DATA_DIR/pdb_chain_enzyme.tsv.gz \

-bc $DATA_DIR/bc-$SEQ_SIM.out \

-seqres $DATA_DIR/pdb_seqres.txt.gz \

-out $DATA_DIR/nrPDB-EC \

printf "\n\n RETRIEVING PDB FILES AND CREATING DISTANCE MAPS...\n"

mkdir $DATA_DIR/annot_pdb_chains_npz/

python PDB2distMap.py \

-annot $DATA_DIR/nrPDB-GO_annot.tsv \

-seqres $DATA_DIR/pdb_seqres.txt.gz \

-num_threads 20 \

-bc $DATA_DIR/bc-$SEQ_SIM.out \

-out_dir $DATA_DIR/annot_pdb_chains_npz/ \

python PDB2distMap.py \

-annot $DATA_DIR/nrPDB-EC_annot.tsv \

-ec \

-seqres $DATA_DIR/pdb_seqres.txt.gz \

-num_threads 20 \

-bc $DATA_DIR/bc-$SEQ_SIM.out \

-out_dir $DATA_DIR/annot_pdb_chains_npz/ \

rm -r obsolete/

printf "\n\n CREATE TFRecord FILES..."

mkdir $TFR_DIR

python PDB2TFRecord.py \

-annot $DATA_DIR/nrPDB-GO_annot.tsv \

-prot_list $DATA_DIR/nrPDB-GO_train.txt \

-npz_dir $DATA_DIR/annot_pdb_chains_npz/ \

-num_shards 30 \

-num_threads 30 \

-tfr_prefix $TFR_DIR/PDB_GO_train \

python PDB2TFRecord.py \

-annot $DATA_DIR/nrPDB-GO_annot.tsv \

-prot_list $DATA_DIR/nrPDB-GO_valid.txt \

-npz_dir $DATA_DIR/annot_pdb_chains_npz/ \

-num_shards 3 \

-num_threads 3 \

-tfr_prefix $TFR_DIR/PDB_GO_valid \

python PDB2TFRecord.py \

-annot $DATA_DIR/nrPDB-EC_annot.tsv \

-ec \

-prot_list $DATA_DIR/nrPDB-EC_train.txt \

-npz_dir $DATA_DIR/annot_pdb_chains_npz/ \

-num_shards 15 \

-num_threads 15 \

-tfr_prefix $TFR_DIR/PDB_EC_train \

python PDB2TFRecord.py \

-annot $DATA_DIR/nrPDB-EC_annot.tsv \

-ec \

-prot_list $DATA_DIR/nrPDB-EC_valid.txt \

-npz_dir $DATA_DIR/annot_pdb_chains_npz/ \

-num_shards 1 \

-num_threads 1 \

-tfr_prefix $TFR_DIR/PDB_EC_valid \

**UNSE**

python protein_distance_matrix_batch.py

-pdb_folder https://alphafold.ebi.ac.uk/

-blast_result_file https://blast.ncbi.nlm.nih.gov/

-fasta_file https://www.uniprot.org/

-fasta_dict

python protein_distance_batch.py

-directory_path

-save_directory

python convert_tsv_to_labels50.py

-tsv_input http://pfam.xfam.org/

python train.py

-multi_label_tsv <http://pfam.xfam.org/>

**MMseqs2**

Creating a database:

mmseqs createdb input.fasta DB

strict clustering:

mmseqs cluster DB DB_clu tmp --min-seq-id 0.8 --cov-mode 1 -c 0.9 --cluster-mode 2

Fast large-scale clustering:

mmseqs cluster DB DB_clu tmp --min-seq-id 0.5 --cov-mode 1 -c 0.7 --cluster-mode 0 --split-memory-limit 20G

Generate readable clustering results:

mmseqs createtsv DB DB DB_clu DB_clu.tsv --threads 16
